# Intestinal helminth infection enhances bacteria-induced recruitment of neutrophils to the airspace

**DOI:** 10.1038/s41598-019-51991-3

**Published:** 2019-10-31

**Authors:** Shao Rong Long, Bernard B. Lanter, Michael A. Pazos, Hongmei Mou, Juliana Barrios, Chien-Wen Su, Zhong Quan Wang, W. Allan Walker, Bryan P. Hurley, Hai Ning Shi

**Affiliations:** 1 Mucosal Immunology and Biology Research Center, Massachusetts General Hospital and Harvard Medical School, Charlestown, MA USA; 2 Department of Parasitology, Medical College of Zhengzhou University, Zhengzhou, Henan China

**Keywords:** Mucosal immunology, Pathogens

## Abstract

Intestinal helminth infections elicit Th2-type immunity, which influences host immune responses to additional threats, such as allergens, metabolic disease, and other pathogens. Th2 immunity involves a shift of the CD4^+^ T-cell population from type-0 to type-2 (Th2) with increased abundance of interleukin (IL)-4 and IL-13. This study sought to investigate if existing gut-restricted intestinal helminth infections impact bacterial-induced acute airway neutrophil recruitment. C57BL/6 mice were divided into four groups: uninfected; helminth-*Heligmosomoides polygyrus* infected; *Pseudomonas aeruginosa* infected; and coinfected. Mice infected with *H. polygyrus* were incubated for 2 weeks, followed by *P. aeruginosa* intranasal inoculation. Bronchial alveolar lavage, blood, and lung samples were analyzed. Interestingly, infection with gut-restricted helminths resulted in immunological and structural changes in the lung. These changes include increased lung CD4^+^ T cells, increased Th2 cytokine expression, and airway goblet cell hyperplasia. Furthermore, coinfected mice exhibited significantly more airspace neutrophil infiltration at 6 hours following *P. aeruginosa* infection and exhibited an improved rate of survival compared with bacterial infected alone. These results suggest that chronic helminth infection of the intestines can influence and enhance acute airway neutrophil responses to *P. aeruginosa* infection.

## Introduction

Acute respiratory infections are the leading cause of pneumonia in children from developing countries^1,2^. Helminths are parasitic worms that have complex lifecycles and are one of the most common infectious agents of humans in developing countries, with children harboring a greater intestinal parasitic worm burden than adolescent and adults^3,4^. Chronic/recurrent intestinal helminth infection and acute infectious pneumonia likely overlap among the pediatric population of developing countries and their potential interrelationship has not been extensively explored

There is a growing interest in understanding how chronic intestinal helminth infections impact host immunity. Helminths are classic inducers of Th2 responses, which reinforce the host capacity to resist and remove large parasitic organisms^5–7^. Th2 cells produce interleukin (IL)-4, IL-5, IL-6, IL-10 and IL-13, which stimulate, both directly and indirectly, the production of IgE, eosinophilia, goblet cell hyperplasia, smooth muscle contraction and changes in the physiology of certain organs^6–8^. While helminth-driven Th2 immunity can be effective at expelling parasites, this immune response appears to be ineffective or potentially pathological in the context of coinfection with additional parasitic, bacterial and/or viral infections^9–16^. In mice and humans, helminth infections correlate with increased severity of tuberculosis, enhanced malaria infection and impaired immunity against *Vibrio cholera*^13,15–20^. Chronic helminth infections can, however, elicit beneficial immunomodulatory effects in the setting of allergic, metabolic, and autoimmune disease^21–25^.

Given pervasive co-infection of respiratory pathogens and intestinal helminths in children from low and middle income countries as well as the complex influence that helminth infection can exert on host immunity, an *in vivo* coinfection model was developed herein to investigate possible influences of chronic helminth infection on bacteria-induced airway inflammation. Specifically, this investigation sought to determine if a chronic gut-restricted *H. polygyrus* infection in mice influenced the early innate immune response within the murine lung challenged with the respiratory pathogen *Pseudomonas aeruginosa*. *Heligmosomoides polygyrus* infection was established in C57BL/6 mice two weeks prior to intranasal challenge with *P. aeruginosa*. Following *P. aeruginosa* infection, lung tissue and airspace contents were examined at 6 hours, and survival was assessed up to 7 days. Importantly, the life cycle of *H. polygyrus* does not involve direct contact with the murine lung thereby facilitating investigation of possible influences of a parasitic gut-restricted mucosal infection on airway responses to *P. aeruginosa*.

Lung infection with *P. aeruginosa* results in rapid recruitment of neutrophils from circulation to lung tissue and airspace^26^. Evidence is provided herein to suggest that coinfection with a gut-restricted *H. polygyrus* impacts neutrophil accumulation specifically within the airspace following airway *P. aeruginosa* infection. Neutrophil recruitment into the airspace is an important feature of acute bacterial pneumonia and these studies as well as the novel model developed, represent a useful approach to investigate a possible interrelationship between intestinal parasitic infection and bacterial pneumonia.

## Results

### Helminth coinfection increased bacterial-induced cellular transepithelial migration into the lung airspace and prolonged host survival

**Figure 1:**
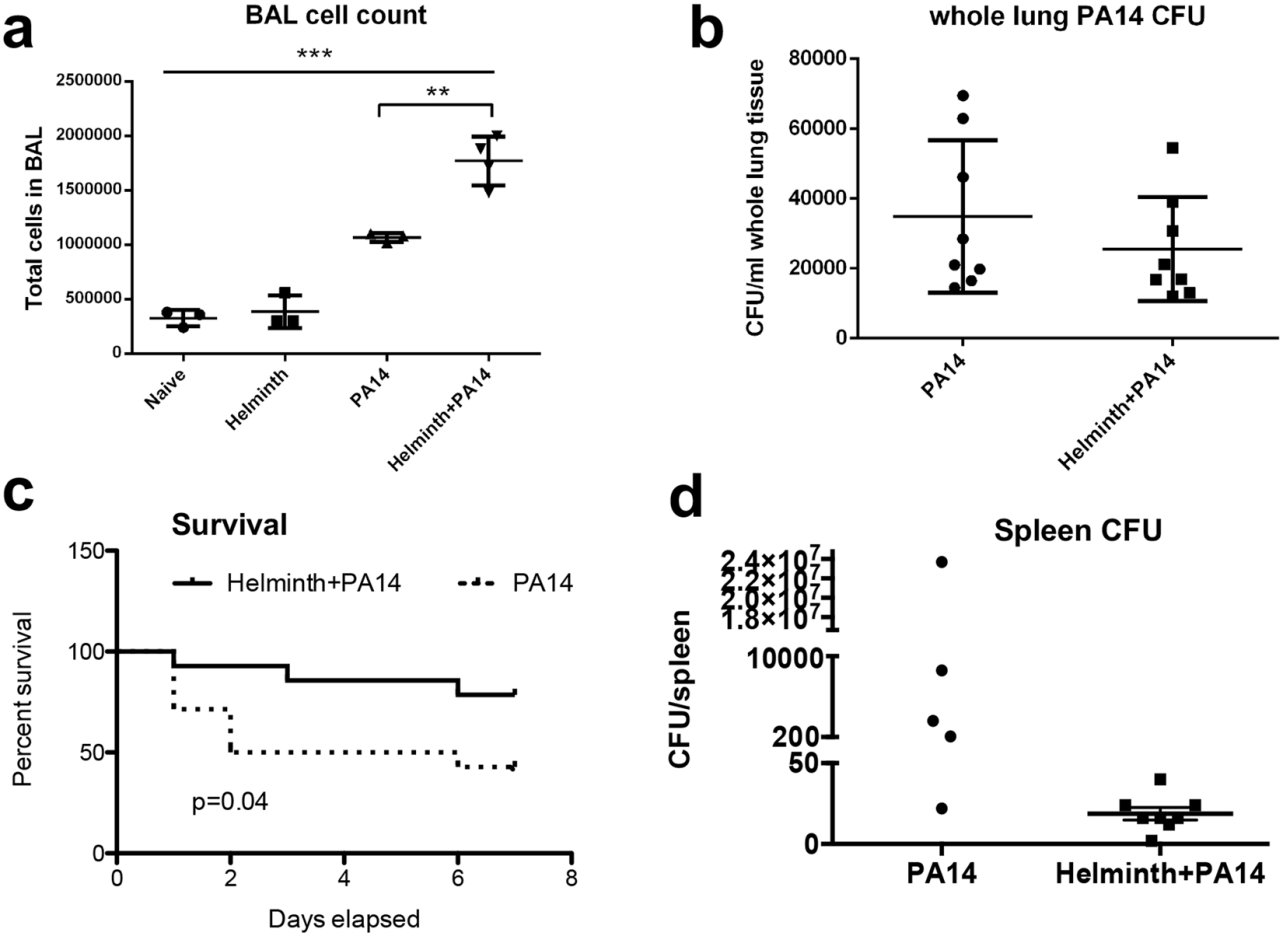
Coinfection with *H. polygyrus* increases cellular transepithelial migration into the lung airspace. After 2 weeks of helminth infection, the mice were infected with/without *P. aeruginosa* through intranasal inoculation. Mice were placed in the cage for 6 hours, and after incubation mice were sacrificed, BAL and lung tissue were obtained. (**a**) The result of BAL cell count. Data are shown as mean ± SD and are representative of three independent experiments. **P < 0.01, ***P < 0.001 between the indicated conditions. (**b**) *P. aeruginosa* CFU in the whole lung tissues without BAL acquisition. Data are shown as mean ± SD, (pooled data, n = 8). (**c**) Coinfection with *H. polygyrus* increases mouse survival and decreases bacterial dissemination into the spleen. After 2 weeks of helminth infection, the mice were infected with *P. aeruginosa* through intranasal inoculation. Mice were placed in the cage and monitored for 7 days. Data shown is the Kaplan Meier survival curve of single infected and coinfected mice over 7 days. Data shown is a compilation of three independent experiments (PA14 n = 14, Helminth + PA14 n = 14). (**d**) *P. aeruginosa* CFU in whole spleen tissues at day 7 post infection. Data are shown as mean ± SEM.

A coinfection model was developed to determine whether an ongoing helminth infection influences the immediate response to infection with *P. aeruginosa* in the airways of mice. After 2 weeks of helminth infection, a random subset of mice from both the naïve group and *H. polygyrus-*infected group were infected with *P. aeruginosa* (strain PA14) through intranasal inoculation as previously described^26^. Mice were placed in the cage for 6 hours and then sacrificed for sample collection and processing as depicted (Supplementary Fig. 1). The total number of cells within bronchial alveolar lavage (BAL) fluid was significantly increased following *P. aeruginosa* infection, as previously observed. Additionally, the total number of cells was increased significantly further in BAL fluid collected from mice coinfected with helminth and *P. aeruginosa*, compared to that detected in mice infected with *P. aeruginosa* alone (Fig. 1a). Despite the significant difference in cellular infiltration of the airspace between bacterial infected and coinfected mice, no significant differences in bacterial burden were observed over a 6-hour bacterial infection period between *P. aeruginosa* infected alone and coinfected mice (Fig. 1b). To further investigate the impact of helminth coinfection on the course of the bacterial infection, we examined animal survival and bacterial dissemination to a peripheral site (spleen) at day 7 post bacterial infection. Our results show that *H. polygyrus* coinfection significantly increased survival rate of infected hosts, which corresponds with a trend showing decreased bacterial dissemination into the spleen (for mice that survived the 7 day period), when compared to *P. aeruginosa* infection alone (Fig. 1c,d).

### Helminth coinfection resulted in increased bacteria-induced neutrophil migration to the lung airspace

**Figure 2:**
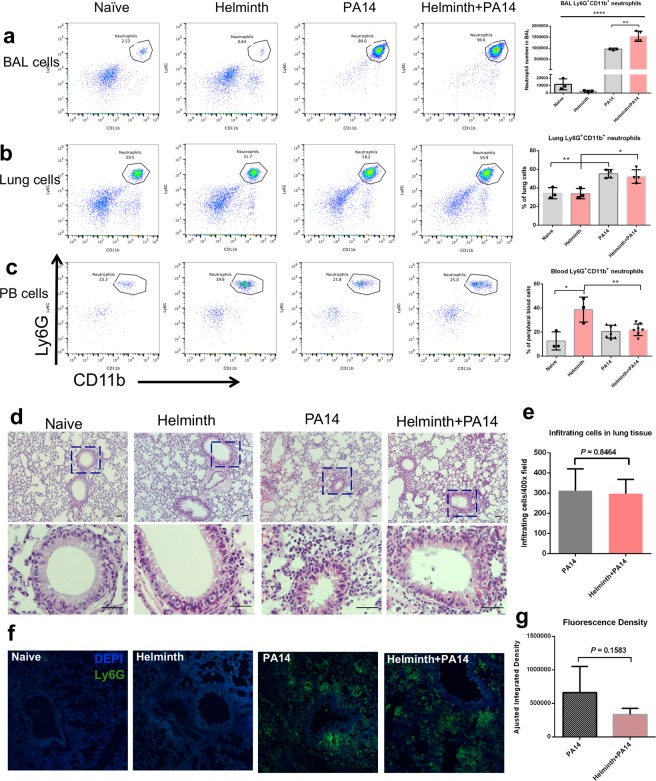
Coinfection with *H. polygyrus* increases neutrophil transepithelial migration into the *P. aeruginosa* infected airspace of the lung. FACS analysis using Ly6G and CD11b markers to isolate the neutrophil population in the (**a**) BAL, (**b**) lung tissue and (**c**) peripheral blood (PB), data are shown as mean ± SD, and are representative of three independent experiments. *P < 0.05, **P < 0.01, ****P < 0.0001 between the indicated conditions. (**d**) The left lung of the mice was stained with H&E, the black bar indicates 20 μm. (**e**) The infiltrating cells in the bronchi, small airways and parenchyma of H&E stained lung sections were counted using ImageJ software. (**f**) Lung sections were stained with FITC-labeled anti-mouse Ly6G. Sections were analyzed by confocal microscopy. (**g**) The fluorescence density of lung sections was enumerated by the ImageJ software (Adjusted Integrated density = Integrated Density-Area*Mean density of the background).

To evaluate the role of intestinal helminth infection in the modulation of initial responses to bacterial infection, we exposed the airway of helminth-infected and control mice to *P. aeruginosa* and examined neutrophil recruitment to the lung at 6 hours post infection. Infection of the lung with *P. aeruginosa* resulted in statistically significant increases in quantities of neutrophils infiltrating the airspace in both the absence and presence of a helminth gut infection when compared to their respective controls; naïve or helminth infection alone (Fig. 2a). Interestingly, there was also a statistically significant increase in the quantity of neutrophils that infiltrated the airspace of coinfected mice when compared to *P. aeruginosa* infection alone (Fig. 2a). Our analysis of neutrophils (Ly6G^+^CD11b^+^) of the lung tissue revealed significantly more neutrophils in coinfected and *P. aeruginosa-*infected hosts compared to their *P. aeruginosa* naïve counterparts (Fig. 2b). No difference in neutrophil counts was detected in whole lung tissue between coinfected and *P. aeruginosa* infected alone mice (Fig. 2b). The overall quantity of neutrophils in circulation 6 hours post infection with *P. aeruginosa* did not appear to be altered in naïve mice, although an increase in circulating neutrophils was noted in helminth alone infected mice, which diminished following *P. aeruginosa* infection (Fig. 2c).

To further determine the impact of intestinal helminth infection on cellular responses in the airway, lung tissue sections were H&E stained. The H&E staining revealed that inflammatory cells infiltrated lung tissue in the presence of *P. aeruginosa* infection (Fig. 2d). However, no significant difference was detected in the cellular infiltration between mice with *P. aeruginosa* infection alone and mice with helminth and bacterial coinfection (Fig. 2e). This is evidenced by the results showing that the similar infiltrating cells in the bronchi, small airways and parenchyma based on H&E stained lung sections were counted using ImageJ software between *P. aeruginosa* infection alone and helminth-coinfection groups (Fig. 2e). Immunofluorescence staining revealed that the similar localization of cells in the lung that were neutrophils (Ly6G^+^) increased in the presence of *P. aeruginosa* lung infection in mice either lacking or containing a parasitic helminth gut infection (Fig. 2f). The quantity of infiltrating cells which were Ly6G^+^ neutrophils in the lung tissue, were not altered by helminth coinfection, when compared to bacterial infection alone (Fig. 2g), consistent with FACS analysis of whole lung tissue described above (Fig. 2b).

### Increased presence of neutrophil-specific enzymes in the airspace of helminth-*P. aeruginosa* coinfected mice

**Figure 3:**
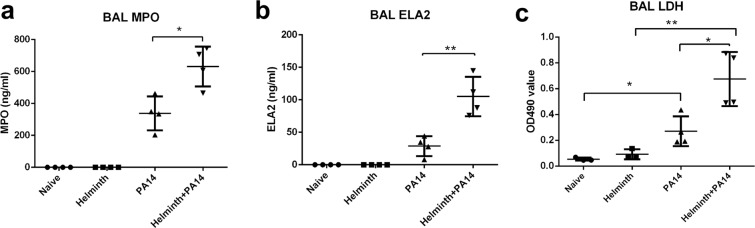
Helminth-induced increase in neutrophil migration during *P. aeruginosa* infection is correlated with increased MPO and ELA2 levels within the airspace. Protein extracts were prepared from BAL samples and analyzed for (**a**) neutrophil myeloperoxidase (MPO) and (**b**) elastase (ELA2). (**c**) LDH levels were assessed in the cell-free supernatant of the BAL to assess cytotoxicity. Data are shown as mean ± SD and are representative of three independent experiments. *P < 0.05, **P < 0.01.

Neutrophils are the dominant cell population recruited during the initial stages of bacterial infection. In our experiments described above, enhanced *P. aeruginosa*-driven neutrophil infiltration specifically into the airspace was observed in coinfected mice (Fig. 2a). To further evaluate this observation, enzymes that are exclusively produced by neutrophils, myeloperoxidase (MPO) and elastase (ELA2), were examined in the BAL fluid of naive mice, mice infected with *H. polygyrus*, mice infected with *P. aeruginosa*, and coinfected mice. MPO (Fig. 3a) and ELA2 (Fig. 3b) were detected only in the airways of *P. aeruginosa* infected mice and the concentration of these enzymes detected in the BAL fluid was significantly increased in the context of coinfection compared to bacterial infection alone. Furthermore, a significant increase in LDH release was observed in the airspace of the coinfected group compared to *P. aeruginosa* infection alone (Fig. 3c), with very little LDH activity observed in the airspace of mice not infected with bacteria. These data further suggest that an existing helminth infection in the intestine correlates with enhanced neutrophil airspace infiltration and possibly activation during *P. aeruginosa* acute infection. Due to their antimicrobial activities, increased early infiltration of activated neutrophils may contribute to an enhanced protection that is observed with the coinfected host (Fig. 1c).

### Gut restricted helminth infection induces goblet cell hyperplasia in the lung tissues

**Figure 4:**
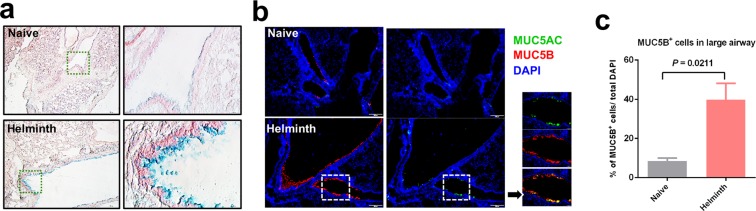
Helminth infection induces goblet cell hyperplasia in the lung tissues. (**a**) Lung sections were stained with alcian blue solution to detect goblet cells. (**b**) The airway was stained with anti-mucin monoclonal antibodies, MUC5AC and MUC5B. (**c**) The quantification of the goblet cells using the ImageJ software, data are shown as mean ± SD.

With the notable finding that mice harboring an intestinal helminth infection displayed a more rapid and robust recruitment of neutrophils to airspace during the initial stages of bacterial lung infection, lung tissue was next examined from mice with and without a gut-restricted helminth infection. It is well established that intestinal helminth infection can significantly increase the quantity of mucosal goblet cells, which synthesize and secrete mucus^6^. Enhanced mucus production has been suggested to impede helminth colonization of the mucosa and promote helminth expulsion from the intestinal tract^27–30^. Whether a gut-restricted intestinal helminth infection influences goblet cell numbers and mucin expression in the airway mucosa has not been explored extensively. Whole lung tissue from naive and helminth-infected mice was collected and examined by immunofluorescence staining to assess goblet cell representation in the airway mucosa. Helminth infected mice display goblet cell hyperplasia when compared to their naive counterparts (Fig. 4a). Further, increased levels of MUC5AC and MUC5B specific mucins were detected in the lung mucosa from mice experiencing chronic infection with gut restricted *H. polygyrus* (Fig. 4b,c). Whether increased mucus and goblet cell hyperplasia impact the neutrophilic response that occurs following infection with *P. aeruginosa* is currently unclear, however, changes within the mucosal airway surface may reflect an alteration of the immunological state of the lung at the time of intranasal bacterial infection due to an ongoing helminth infection in the gut.

### Gut restricted helminth infection promotes increased CD4^+^ T-cells in the airspace

**Figure 5:**
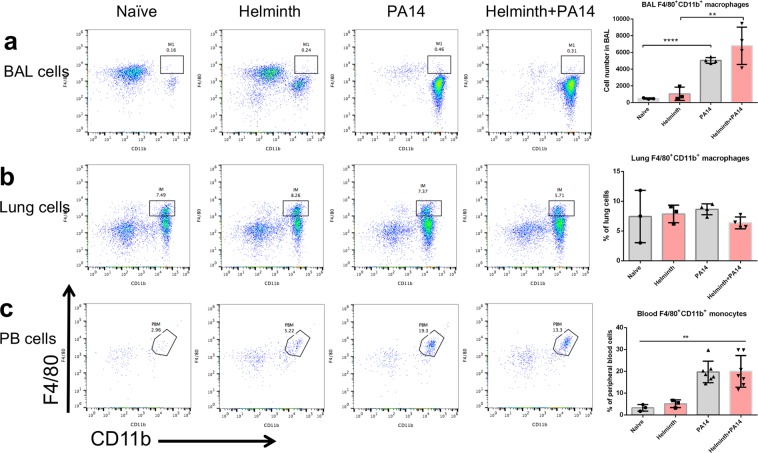
Coinfection with *H. polygyrus* does not impact macrophage levels within the airspace, lung tissue or circulation during *P. aeruginosa* infection of the airspace. FACS analysis using F4/80 and CD11b markers to isolate the macrophage populations in the (**a**) BAL, (**b**) lung tissue and (**c**) monocytes in peripheral blood. Data are shown as mean ± SD and are representative of three independent experiments. *P < 0.05, **P < 0.01, between the indicated conditions.

**Figure 6:**
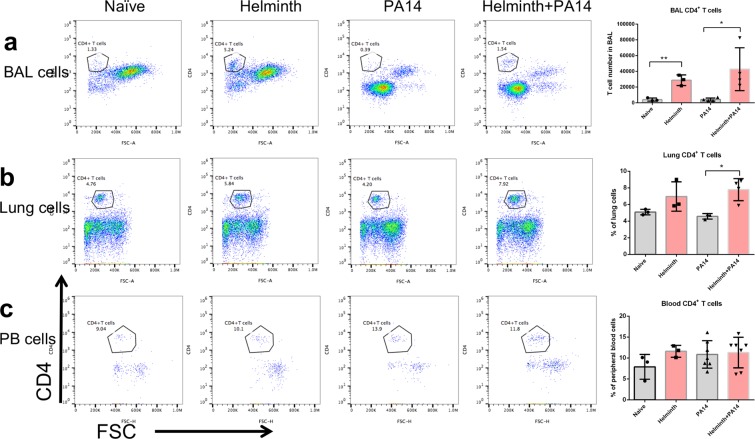
Coinfection with *H. polygyrus* during *P. aeruginosa* acute pneumonia increases the CD4^+^ T-cells population in the airspace and lung tissues. FACS analysis using CD4 marker to isolate the CD4^+^ T-cell population in the (**a**) BAL, (**b**) lung tissue and (**c**) peripheral blood. Data are shown as mean ± SD and are representative of three independent experiments. *P < 0.05, **P < 0.01 between the indicated conditions.

Helminth infection did not exert any significant impact on monocytes/macrophage populations in the airspace, lung tissue, or circulation (Fig. 5), although bacterial infection did significantly increase airspace macrophages (Fig. 5a) and monocytes in circulation (Fig. 5c). In contrast, helminth infection elicited higher quantities of CD4^+^ T-cells within the airspace of the lung when compared to naïve mice irrespective of bacterial infection (Fig. 6a). A similar trend was observed regarding the percent of CD4^+^ T-cells found in lung tissue, although in mice not receiving a bacterial lung infection, the percent of CD4^+^ T-cells derived from lung tissue of mice infected with helminth alone did not increase to a statistically significant degree compared to naive mice (Fig. 6b). The quantity of CD4^+^ T-cells in circulation did not appear to be altered in any context (Fig. 6c). In addition, the frequency of eosinophils, a cell population that can be induced by helminth infection and is associated with a Th2 immune response, was increased significantly in BAL fluid and lung tissue collected from mice coinfected with helminth and *P. aeruginosa*, compared to that detected in mice infected with *P. aeruginosa* alone (Supplementary Fig. 2).

### Expression of inflammatory mediators in the lungs of naive, singly and coinfected mice

**Figure 7:**
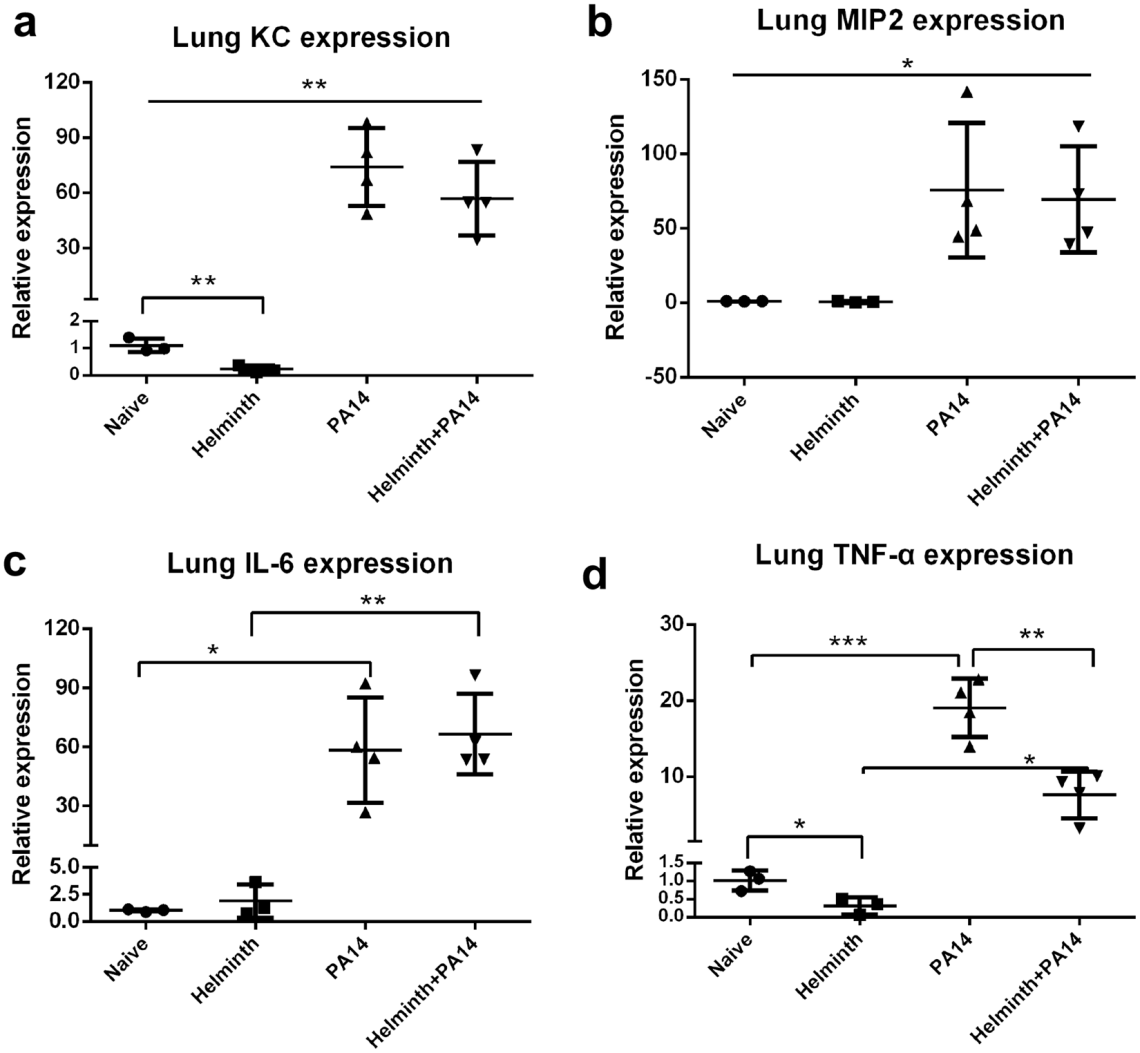
Prior enteric helminth infection has differential impact on inflammatory mediators. (**a–d**) Lung tissues were collected from naïve, *H. polygyrus*-infected, PA14-infected, and helminth-coinfected mice. Total RNA was isolated from the lung tissues. *KC, MIP2, IL-6* and *TNF-α* expression was determined using RT-qPCR. Values are the relative expression compared to the naïve mice. The data shown are means ± SD from one of three experiments performed showing similar results. *P < 0.05, **P < 0.01, ***P < 0.001.

The presence of an intestinal helminth infection during *P. aeruginosa* airway infection resulted in augmented neutrophil transepithelial migration into the airspace of the infected lung (Figs 2a and 3). Inflammatory factors and chemokines play an important role in neutrophil recruitment^31–33^. Expression of *KC* (CXCL1), *MIP2* (CXCL2), *IL-6*, and *TNF-α* were evaluated by qPCR in lung tissue. Helminth infection in the intestine reduced the *KC* lung expression in the absence of *P. aeruginosa* infection, however, during *P. aeruginosa* infection of the lung, *KC* expression levels were increased and similar in the presence or absence of a concomitant helminth infection (Fig. 7a). The expression levels of *MIP2, IL-6 and TNF-α* in the lung tissue was also significantly upregulated following *P. aeruginosa* infection irrespective of helminth coinfection (Fig. 7b–d). Interestingly, *TNF-α* lung expression was suppressed in the presence of helminth infection when compared to respective naive or *P. aeruginosa*-infected mice (Fig. 7d).

### Expression levels of Th2 mediators in naive, singly and coinfected mice

**Figure 8:**
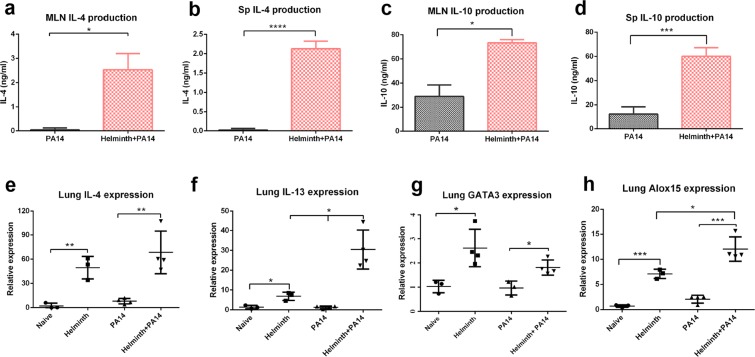
Helminth coinfection promotes Th2 cytokine expression and production, resulted in upregulation of *Alox15* expression in the lung tissue. (**a–d**) Mediastinal lymph nodes (MLNs) and spleen (Sp) cells were collected from the mice in the presence of *P. aeruginosa* infection and stimulated *in vitro* with anti-CD3 mAb. Culture supernatants were collected 72 h later. Cytokine (IL-4 and IL-10) secretion into the culture supernatants was determined by ELISA. (**e–h**) Total RNA was isolated from the lung tissues. *IL-4, IL-10, GATA3* and *Alox15* expression was determined using RT-qPCR. Values are the relative expression compared to the naïve mice. The data shown are means ± SD from one of three experiments performed showing similar results. *P < 0.05, **P < 0.01, ***P < 0.001, ****P < 0.0001.

Helminth intestinal infection promotes Th2 immunity, which modulates the host cytokine expression profile to defend against a parasitic infection^5,6^. At 6 hours post bacterial infection, we separated mediastinal lymph nodes (MLNs) and spleens from coinfected and *P. aeruginosa* infected mice, isolated lymphocytes, and stimulated them with anti-CD3 to evaluate cytokine production. Lymphocytes from both MLNs and spleen of mice with a preexisting enteric helminth infection displayed significantly enhanced release of IL-4 and IL-10 (Fig. 8a–d), indicating a Th2-dominated immune response occurring systemically.

Since helminth infected mice displayed evidence of systemic Th2 immunity, gene expression levels of Th2 cytokines *IL-4* and *IL-13* were examined in the lung. Helminth infection in the intestine was associated with a significant upregulation of *IL-4* and *IL-13* expression in lung tissue (Fig. 8e,f). Coinfected mice had significantly higher *IL-13* expression within lung tissues when compared with helminth infection alone (Fig. 8f). These observations are in line with our results showing the detection of the helminth-induced goblet cell hyperplasia, which is typically seen as part of a Th2 response (Fig. 4) and increased frequency of eosinophils in the lung tissues (Supplementary Fig. 2). Furthermore, gene expression level of GATA3, the master transcription factor of the Th2 response, was examined. Similarly, helminth infection was associated with a significant upregulation of *GATA3* expression in the lung tissue (Fig. 8g). These results demonstrate that an intestinal helminth infection promotes the development of Th2 immune response in lung. Both *IL-4* and *IL-13* have been shown to increase the transcription and activity of the lipoxygenase gene *Alox15*^34,35^, which encodes an enzyme capable of generating inflammatory lipid mediators, including hepoxilin A3^36^. Hepoxilin A3 (HxA_3_), is an eicosanoid chemotactic mediator that drives the migration of neutrophils across mucosal epithelial barriers into the airspace in the context of bacterial infection^37–40^. Accordingly, the impact of helminth infection on *Alox15* was determined. The results from qRT-PCR analysis revealed that gut-restricted helminth infection resulted in increased expression of *Alox15* in lung tissue when compared with naive mice (Fig. 8h). Interestingly, helminth coinfection induced a significantly greater level of *Alox15* expression in lung tissue when compared with either *P. aeruginosa* infection alone or helminth infection alone (Fig. 8h).

## Discussion

Pneumonia is the leading cause of death amongst children globally. In 2010, the World Health Organization (WHO) reported 0.22 episodes per child-year of community-acquired pneumonia among low- and middle-income countries and approximately 11.5% of these cases progress to severe episodes^2,41^. It is likely that a large subset of children with community-acquired pneumonia are also infected with helminths as billions of people worldwide currently host parasitic helminth infections. Helminth infections are also widespread in low- and middle-income countries with poor hygienic conditions. Although direct pathological damage by helminth is an important aspect to morbidity, the modulatory role of the parasite on the host immune system has additional implications to human health^11,42,43^. Chronic helminth infection results in the manipulation of host immune responses, skewing the host towards type 2 immunity. Type 2 immunity includes innate and adaptive components, characterized by increases in the levels of type 2 cytokines (including IL-4, IL-5, IL-9, and IL-13), activation / expansion of type 2 T cells (Th2) and innate lymphoid cells (ILC2), and recruitment of eosinophils, mast cells, basophils, and alternatively activated macrophages^44–46^.

Helminthic and bacterial infections coexist in many parts of the world, and helminth-induced Th2 immunity can alter the host response to bacterial pathogens. Experimental animal models showed that infection of mice with the small intestinal helminth parasite *H. polygyrus* exacerbates infection and disease caused by concurrent infection in the colon with enteropathogenic bacteria via a Th2-dependent mechanism^10,11^. Mice with a persistent helminth-mediated type 2 immunity are less effective at responding to a chronic mycobacteria infection and display a higher bacterial burden in the lung. The immune response to mycobacteria infecting the lung is characterized by an increase in Th2-dominated cytokines with associated development of alternatively activated macrophages and suppression of Th1 and Th17 responses in the context of coinfection with helminthes^13^. Chronic infection with the murine helminth *H. polygyrus* can, however, mediate protection against allergic asthma by eliciting a regulatory T cell population^24^ and/or altering the intestinal microbiota resulting in increased short chain fatty acid production^25^. In a recent report, Th2 immune responses, including IL-5 and recruited activated eosinophils, induced by infection with the intestinal helminth parasite, *H. polygyrus*, has been shown to promote host protective response to the lung migratory parasite, *Nippostrongylus brasiliensis*^47^. Evidence also indicated that the strictly enteric *H. polygyrus* infection can promote Th2 cell spread systemically into the liver, airways, and peritoneal cavity^48^. Enteric *H. polygyrus* infection can exert protective antiviral effects in the lung through induction of a microbiota-dependent type I interferon response^14^. However, it is unclear whether and how helminth infection and helminth-induced Th2 immune responses affect respiratory bacterial infection from organisms such as *P. aeruginosa* that can cause bacterial pneumonia. One hypothesis that we tested in the current study was that an intestinal helminth infection may modulate bacteria-induced neutrophil responses in the airway. We found that mice harboring a helminth infection in the gut exhibited a more rapid and robust infiltration of neutrophil into the airspace following airway infection with *P. aeruginosa* compared with mice lacking any helminth infection. Furthermore, we observed a significant increase in survival among mice harboring helminth compared to mice with *P. aeruginosa* infection alone. Additionally, mice that survived 7 days following *P. aeruginosa* infection, yet lacked a helminth coinfection, trended toward having more bacteria in the spleen suggesting that enhanced bacterial dissemination may have occurred in mice lacking a gut-restricted helminth infection. Whether the augmented neutrophil recruitment to the airspace observed during the initial stages of bacterial airway infection of mice coinfected with enteric helminth contributes to the improved survival and reduced bacterial dissemination, remains to be elucidated.

An *in vivo* coinfection model system involving enteric infection with *H. polygyrus* to establish chronic murine helminthiases^30^, followed by airway administration of a lung bacterial pathogen (*P. aeruginosa*)^26^, was developed and characterized to analyze the effects of chronic intestinal helminth parasitic infection on the initial stages of bacterial infection of the airway. Our results show that mice co-infected with helminth and bacteria (PA14) have an enhanced Th2 response in both lung and systemic sites and that the increased Th2 response (GATA3, IL-4, IL-13 and eosinophils) detected in lung, was associated with an enhanced Alox15 expression in coinfected mice. Our results further show that helminth coinfection resulted in augmented *P. aeruginosa*-induced neutrophil transepithelial migration into the airspace, which was detected through FACS analysis of Ly6G^+^CD11b^+^ cell populations within the BAL fluid. These data were further supported by the increased levels of neutrophil myeloperoxidase and elastase within the airspace.

Recruitment of neutrophils into lung tissue during bacterial infection plays a vital role in controlling bacteria at early stages of the infection. However, excessive neutrophil infiltration characterized by substantial neutrophilic breach of the airway mucosa resulting in accumulation of neutrophils in the airspace contributes to damaging host-derived inflammation and tissue injury. Our observations suggest that concurrent intestinal helminth infections increase the magnitude of airspace neutrophil recruitment at the early stages of bacterial lung infection. Chemokines play an important role in the movement and localization of inflammatory cells in disease^31,32^. We examined whether a concurrent parasitic helminth infection may augment expression of two major neutrophil chemoattractants, KC and MIP2, in lung tissues. Analysis by qPCR demonstrated the presence of *P. aeruginosa* infection induced expression of *KC* and *MIP2* within the lung tissue, which correlated with the increased neutrophil infiltration of infected lung tissue. However, the presence of helminth coinfection did not impact the number of neutrophils entering the lung tissue nor the expression level of these two chemokines, believed to be responsible for driving neutrophils out of circulation. Interestingly, an increase in expression of *Alox15* was observed to be associated with helminth infection and expression was increased further in the context of coinfection with *P. aeruginosa*. The enzyme encoded by *Alox15* is a lipoxygenase with both 12- and 15-lipoxygenase activity^38,49,50^, an enzyme previously demonstrated to be involved with driving neutrophil transepithelial migration^51,52^. Since neutrophil transepithelial migration is necessary for emigration of neutrophils into the airspace, it is interesting that both *Alox15* expression and airway neutrophil accumulation are coordinately increased during coinfection. Furthermore, IL-4 and IL-13 are known to upregulate transcription and activity of *Alox15*^34,35^ and expression of these Th2 cytokines correlates with *Alox15* expression during coinfection in the study herein. Whether Alox15 is responsible for increased neutrophil accumulation in the airspace of coinfected mice is an important question that remains to be elucidated.

Helminths are potent regulators of the host’s immune system. It has been demonstrated that intestinal goblet cell hyperplasia develops in response to intestinal helminth infections, and the observed hyperplasia seems to be dependent on a functional T-cell response^29^. However, it was unclear if gut-restricted helminth infections had the capacity to impact distal organs and promote goblet cell hyperplasia within lung tissues potentially through helminth-induced Th2 associated cytokines produced beyond the intestine. In our present study, we observed that helminth infected mice had significantly increased number of CD4^+^ T-cells in the airspace when compared to naive mice, as well as enhanced lung expression of Th2 cytokine genes *Il-4* and *Il-13*. These results are supported by previous report showing *H. polygyrus* infection promote Th2 cell localization into distal sites, including the lung airways^48^. Our histological analysis revealed goblet cell hyperplasia in the lung tissues, which correlated with increased levels of MUC5AC and MUC5B production in the lung mucosa. In the airways, MUC5AC and MUC5B are secreted mucins that have direct antimicrobial activity and the ability to trap and mediate swift removal of inhaled pathogens by mechanical clearance^53,54^. Whether *P. aeruginosa* encountering an altered airway mucosa resulting from the presence of helminths in the gut has an influence on the neutrophilic response in the lung remains to be determined and can be examined using this newly developed coinfection model in future studies.

In summary, our observations suggest that a pre-existing parasitic gut infection enhances infiltration of neutrophils into the airspace in the early stages of *P. aeruginosa* airway infection and promotes the survival of the co-infected host, likely through helminth-orchestrated systemic Th2 immunity. The establishment of our model demonstrating enhanced airspace neutrophil infiltration during coinfection, will be leveraged in future experiments to characterize mechanistic underpinnings of this important observation. We hypothesize that these mechanisms may be relevant regarding the prevalence and severity of pneumonia in developing countries and chronic helminth infections may be an important modulating factor worthy of epidemiological investigation. With this model we begin to characterize the effects of helminth infection on the initial stage of bacterial infection of the airway. Future studies utilizing targeted mouse gene deletion approaches will provide more definitive insight into the molecular mechanisms by which helminth infection and helminth-induced Th2 response regulate *P. aeruginosa* infection, airway inflammation and disease. Nevertheless, this study establishes a novel coinfection model and provides evidence for an altered immune response to a bacterial pathogen infecting a distinct organ in the context of hosting a chronic gut-restricted helminth infection. Further, we characterize the cellular nature of the altered immune response and present changes in gene expression that may be associated with this altered cellular response.

## Methods and Materials

### Bacterial strain

All bacterial infections were carried out with *Pseudomonas aeruginosa* strain PA14. Bacterial cultures were grown as previously described in LB medium at 37 °C, 220RPM^26^.

### Mice

Pathogen-free, 6-week-old female C57BL/6 mice were purchased from the Jackson Laboratory (Bar Harbor, ME). All animal studies were carried out in accordance with the recommendations in the Guide for the Care and Use of Laboratory Animals of the National Institutes of Health. The protocol was approved by the Sub-committee on Research Animal Care of Massachusetts General Hospital (Animal Welfare Assurance Number A3596–01).

### Helminth infection

The helminth parasite *H. polygyrus* was cultivated as previously described^11^. Mice were randomly separated into two groups; the helminth infection group was administered 200 third-stage larvae (L_3_) orally by gavage with a 21-gauge feeding needle. *H. polygyrus* infection was incubated for 2 weeks prior to infection of the mice with *P. aeruginosa*.

### Intranasal bacterial innoculation

Infection of the mouse airway with *P. aeruginosa* was carried out as previously described^26^. After 2 weeks of helminth infection, a random subset of 7 mice from both the naïve group and *H. polygyrus-*infected group were infected with *P. aeruginosa* (strain PA14) through intranasal inoculation (45 μl, 1.3e^7^ CFU/mL). The groups that were not infected with bacteria were inoculated intranasally with the same volume of sterile HBSS (45 µL) as mice receiving PA14 infection. After intranasal inoculation, the mice were placed in the cage for 6 hours, at which time the mice were euthanized and samples were obtained and processed.

### Processing of bronchial alveolar lavage

Bronchial alveolar lavage (BAL) samples were obtained and processed as previously described^26^. Lungs were flushed with 2 mL total (0.5 mL per wash, 4×) of HBSS supplemented with 0.3% FBS and 300 μM EDTA. Collected BAL was separated into three separate aliquots: 1.0, 0.5, and 0.5 mL. Cell count and viability measurements using trypan blue stain were performed using Countess Automated Cell Counter (Invitrogen). Cells were blocked with anti-CD16/CD32 (BD Biosciences, San Jose, CA) for 20 min on ice and processed for flow cytometry. For protein lysate samples, one of the 0.5 mL BAL aliquots was treated with 10 μL protease inhibitor cocktail set III, EDTA-free (Calbiochem, MA) and 20 μl of 10% Triton X-100 (Sigma, MA) and mixed by inversion for 20 minutes at 4 °C. Protein lysate samples were used for mouse neutrophil elastase/ELA2 and MPO ELISA (R&D Systems, MN) per manufacturers protocol. The other 0.5 mL BAL aliquots were centrifuged, and cell-free supernatant was collected and used for lactate dehydrogenase (LDH) release assay according to manufacturer’s instructions (Sigma).

### Bacterial recovery from mouse lungs

Bacterial recovery was carried out as previously described^26^. Whole lung tissue, which BAL was not acquired from, was removed surgically and placed into 2 mL Eppendorf tubes which contained a 5 mm metal bead with 1 mL of HBSS + and stored on ice. Tubes were placed in the TissueLyser LT (Qiagen, Germany, 50 Hz, 6.5 min). Lung homogenate were plated on *Pseudomonas* isolation agar plates using the drop plate method and grown at 37 °C overnight. Colonies were counted, and the total CFU/mL recovered from the homogenized tissue samples was calculated.

### 7 days of bacterial infection

C57BL/6 mice were randomly separated into two groups: bacterial infection alone and helminth coinfection groups. For the coinfection mice, *H. polygyrus* infection was incubated for 2 weeks prior to infecting  the mice with *P. aeruginosa*. Mice were infected with *P. aeruginosa* (strain PA14) through intranasal inoculation (45 μl, 1.3e7 CFU/mL). After intranasal inoculation, mice were placed in the cage and monitored, the status of mice was observed each day until day 7, when mice were euthanized and the spleen was collected. Spleen homogenate were plated on *Pseudomonas* isolation agar plates and grown at 37 °C overnight. Colonies were counted, and the total CFU recovered was calculated.

### Acquisition and preparation of samples for qRT-PCR

Total RNA was prepared from lung tissues using TRIzol reagent (Invitrogen Life Technologies) following the manufacturer’s recommendations and reverse transcribed into cDNA using a PTC-200 Peltier Thermal Cycler (MJ RESEARCH). The cDNA samples were then tested for the expression of *KC, MIP2, IL-4, IL-6, IL-13, GATA3, TNF-α*, and *Alox15* by real-time quantitative PCR using SYBR Green PCR Master Mix (Apex Bioresearch Product) on a StepOne Plus Real-Time PCR system (Applied Biosystems). GAPDH was used as the housekeeping control. The gene expression level was normalized by subtracting the expression level of GAPDH of the same group, and the different expression levels were calculated using the comparative Ct (2^−∆∆Ct^) method.

### Lymphocyte isolation and T-cell cytokine production measurement

Spleens and mediastinal lymph nodes (MLNs) from *P. aeruginosa* infected mice were harvested and placed in complete Dulbecco’s modified Eagle’s medium (cDMEM). Lymphocyte suspensions were prepared from the MLNs and spleens by pressing the cells through a 70-μM nylon cell strainer (Falcon; BD Labware). Red blood cells were lysed. The cell number was counted using the Countess Automated Cell Counter. Cells (5 × 10^6^ cells/ml) were cultured in a 48-well plate pre-coated with anti-CD3 monoclonal antibody ((BD Pharmingen, 5 μg/ml), and culture supernatants were collected after 72 h of stimulation. Culture supernatants were analyzed for the levels of cytokines present via ELISA as previously described^11^.

### Tissue harvest, sectioning, and goblet cell characterization

Mice were euthanized with carbon dioxide gas. Lung tissues were harvested and fixed at 4 °C overnight with fresh 4% paraformaldehyde. The tissues were then rinsed and incubated in 30% sucrose in PBS at 4 °C overnight. Afterwards, the tissues were soaked in Tissue-Tek® O.C.T. Compound for 1 hour and then frozen in O.C.T. for cryosectioning at 7μm thickness. The slides were stained with primary antibody for 2 hours at room temperature. Following incubation, the slides were rinsed and incubated with secondary antibodies at room temperature for 1 hour. After rinsing 4 times, the slides were stained for DAPI (Sigma) for 1 minute and mounted with Fluromount-G medium (SouthernBiotech). The antibodies used were MUC5AC (Thermo Scientific) and MUC5B (Sigma). The slides were also stained with alcian blue solution to detect goblet cells. The slides were first stained for 10 min with 1% alcian blue solution in 3% acetic acid, pH 2.5 (Poly Scientific). Then slides were rinsed under tap water for 1 min and then were counter-stained for 1 minute with 0.1% Nuclear Fast Red kernechtrot (Poly Scientific). Afterwards the slides were briefly dried and mounted in Fluromount-G medium. The staining on slides was visualized using Olympus IX81 inverted fluorescence microscope. Images were captured and combined using MicroSuite FIVE (Olympus Soft Imaging Solutions) and Extended Focal Imaging (EFI) module to create a single in-focus image. To quantify the goblet cells, the acquired images from the fluorescence microscope were processed using the ImageJ software. The quantification was performed by counting at least 5 random fields of view with 40x objective and calculating the average and standard deviation.

### Histology examination

At necropsy, left lung tissues were collected, formalin-fixed and embedded in paraffin. The processed tissues were sectioned into 5μm thick slices and stained with hematoxylin and eosin (H&E) to assess the pathology. The quantification of infiltrating cells was performed by ImageJ

### Immunofluorescence detection of neutrophils

The slides were blocked with avidin/biotin agent (Vector Laboratories, Burlingame, CA, USA). To analyze the abundance of neutrophils, lung tissue slides were stained with FITC-labeled anti-mouse Ly6G (Biolegen, 1:200). Nucleuses were stained and mounted using DAPI solution (Vector Laboratories). Sections were analyzed by microscopy and the quantification was performed by ImageJ.

### Flow cytometry preparation and analysis

To prepare the lung tissues for flow cytometry, they were first cut into small pieces and incubated with 1 mg/ml collagenase D (Worthington) and 0.5 mg/ml DNAse I (Roche) at 37 °C for 30 mins. Collagenase was inactivated with 1 ml sterile DMEM containing 10% FBS; the digested tissues were transferred to a 70-μM nylon cell strainer and disrupted using a syringe plunger to obtain single cell suspensions. Following lung tissue processing, the resulting single cell suspension, BAL, and peripheral blood samples were processed in 1 ml Red Blood Cell Lysing Buffer (Sigma). The cellular populations of the lung tissue, BAL, and peripheral blood from different groups were identified through the following markers: neutrophils were identified with anti-Ly6G (Biolegend) and CD11b (abcom) monoclonal antibody (mAb), macrophages/monocytes were identified with anti-F4/80 (eBiosciencce) and CD11b mAb, T cells were identified with anti-CD4 mAb (BD Pharmingen), eosinophils were identified with anti-siglecF mAb. The stained samples were analyzed with an Attune NxT Acoustic Focusing Cytometer (Thermo Fisher). Dead cells and debris were excluded from analysis via size exclusion.

### Statistical analysis

All results were expressed as the mean ± SD. Statistical differences were determined using a two-tailed Student t test with GraphPad Prism. A *P* value < 0.05 was considered significant.

## Supplementary information

Supplementary Figure 1 and 2
