# Supplementary material for: Intestinal helminth infection enhances bacteria-induced recruitment of neutrophils to the airspace
Source: Sci Rep. 2019 Oct 31;9:15703. doi: 10.1038/s41598-019-51991-3 (PMC6823376; doi:10.1038/s41598-019-51991-3)
Supplement: Supplementary file 1 — Supplementary Figure 1 and 2 [file 41598_2019_51991_MOESM1_ESM.pdf]

Intestinal helminth infection enhances bacteria-induced recruitment of neutrophils to the airspace

Shao Rong Long[1, 2¶], Bernard B. Lanter[1 ¶], Michael A. Pazos[1], Hongmei Mou[1], Juliana Barrios[1], Chien-Wen Su[1], Zhong Quan Wang[2], W. Allan Walker[1], Bryan P. Hurley[1, ¥, *], Hai Ning Shi[1, ¥, *]

Supplementary Figure 1

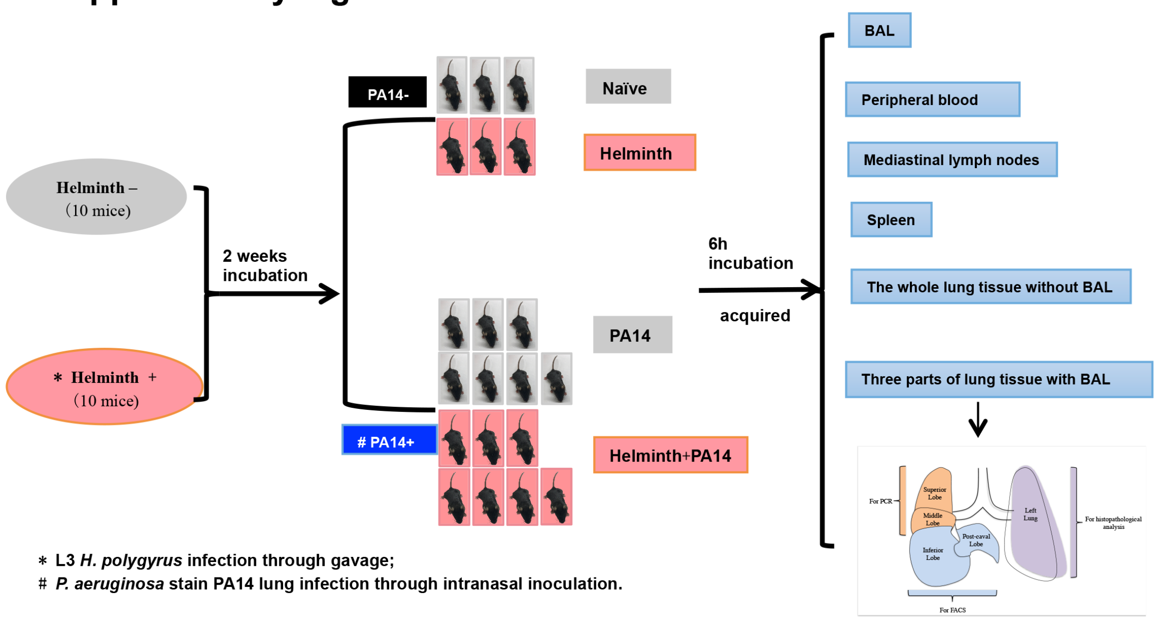

Supplementary Figure 1. Schematic of the helminth- *P. aeruginosa* coinfection experimental process, run as three independent experiments. 20 mice were randomly separated into two groups; naïve and *H. polygyrus* helminth infected, which were administered 200 third-stage larvae (L3) orally by gavage. After 2 weeks of helminth

infection, a random subset of 7 mice from both the naïve group and *H. polygyrus*-infected group were infected with *P. aeruginosa* through intranasal inoculation (45 μl, 1.3e^{7} CFU/mL). Mice were set as 4 groups according to the infection-state; Naïve-no infection, Helminth- *H. polygyrus* infection alone, PA14- *P. aeruginosa* infection alone, Helminth+PA14- *H. polygyrus* and *P. aeruginosa* coinfection. Mice were placed in the cage for 6 hours, and after incubation mice were sacrificed and either samples of BAL, peripheral blood, mediastinal lymph nodes, spleen and lung tissues were obtained or the whole lung tissue was harvested and homogenized for bacterial burden assessment.

Supplementary Figure 2

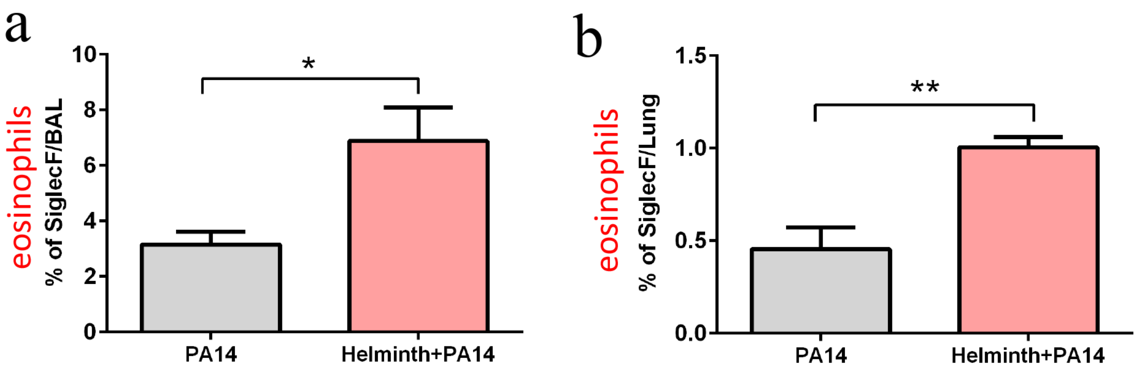

Supplementary Figure 2. Coinfection with *H. polygyrus* during *P. aeruginosa* acute pneumonia increases the siglecF^{+} eosinophil population in the airspace and lung tissues. FACS analysis using siglecF marker to dtecte the siglecF^{+} eosinophil population in the **(a)** BAL and **(b)** lung tissue. Data are shown as mean ± SD, and are representative of two independent experiments. *P<0.05, **P<0.01 between the indicated conditions.
